# Dynamic Cone Penetrometer Incorporated with Time Domain Reflectometry (TDR) Sensors for the Evaluation of Water Contents in Sandy Soils

**DOI:** 10.3390/s19183841

**Published:** 2019-09-05

**Authors:** Won-Taek Hong, Jung-Doung Yu, Sang Yeob Kim, Jong-Sub Lee

**Affiliations:** 1 Department of Civil and Environmental Engineering, University of Illinois at Urbana-Champaign, 205, North Mathews Avenue, Urbana, IL 61801, USA; 2 School of Civil, Environmental and Architectural Engineering, Korea University, 145, Anam-ro, Seongbuk-gu, Seoul 02841, Korea

**Keywords:** dynamic cone penetrometer, penetration index, relative permittivity, time domain reflectometry, volumetric water content

## Abstract

Ground moisture content and strength properties are the most important factors for a proper assessment of ground stability. This study developed a dynamic cone penetrometer incorporated with time domain reflectometry (TDR) sensors (TDCP). The TDCP is composed of an anvil, a driving rod, and a TDCP probe. Three wave guides and a K-type thermocouple are installed on the TDCP probe. For utilization of TDCP, relationships between relative permittivities measured by TDCP and those measured by standard TDR probe, temperature, and volumetric water content of the soils were investigated. In addition, the relationship between penetration indices by TDCP (TPI) and by standard dynamic cone penetrometer was established. In the field application test, relative permittivity, ground temperature, and TPI were measured along the depth. Moreover, gravimetric water contents were also measured for comparison. The experimental results showed that volumetric water contents compensated by ground temperature showed good agreement with the volumetric water contents estimated from the gravimetric water contents of the soil samples and TPI. This study suggests that the TDCP may be effectively used for the evaluation moisture contents and for the strength characterization of the subsurface.

## 1. Introduction

Most subsurface in shallow depths is composed of air-dried and unsaturated soils. Under general circumstances, subsurface soils are in steady states. However, drastic changes of water contents in the soil mixture can cause anomalous behavior of the subsurface [1,2]. The increase of the unit weight of soils due to heavy rain causes excessive shear stress in the ground. The rise of the subsurface water level decreases the shear strength of the subsurface ground. The excessive shear stress and the decrease in the shear strength of the soils can result in the shear failure of the slope, such as landslide and failure of embankment [3,4,5,6]. In addition, fracture or breakage of underground pipes in urban areas can generate unexpected subsurface water flow that causes soil erosion and decreases shear strength [7,8,9]. To ensure subsurface stability, subsurface water content and ground strength should be properly evaluated. However, because sloping ground, such as embankment and mountainous ground, is poorly accessible and urban ground is limited for ground excavation, an efficient method that can estimate subsurface water contents and ground strength with a high portability and minimal ground disturbance is needed.

A dynamic cone penetrometer (DCP) is a miniaturized in-situ penetration testing apparatus that can estimate ground strength by penetration index [10]. The DCP is suitable for near-surface characterization of the slope owing to its high portability and simplicity [11]. To expand the applicability of DCP, relationships between penetration index and the other engineering parameters such as stiffness, void ratio, relative density, and California bearing ratio have been studied [12,13,14]. However, water content cannot be estimated from the penetration index. Thus, an instrumentation of DCP is required for effective estimation of the subsurface water contents. To estimate the soil–water characteristics in geotechnical, geo-environmental, and agricultural practices, soil tensiometer and time domain reflectometry (TDR) are commonly used [15,16]. Soil tensiometer determines the matric potential of the soil by reading the pressure of the vacuum gauge which indicates the water flow between the tube and soils surrounding the tensiometer. On the other hand, the TDR measures the relative permittivity of the ground which is contacted with a probe and evaluates the volumetric water content based on relative permittivity [17,18,19]. For field application of soil tensiometer and TDR, pre-boring or pre-excavation is required because when penetrating the ground, probes used in those methods can be easily broken. Thus, soil tensiometer and TDR have been commonly used for ground surface or small-scale laboratory tests [20]. In particular, it is hard to incorporate the soil tensiometer into the DCP because the soil tensiometer requires installation of water filled pipe and porous ceramic cup [21] which are prone to being fractured or clogged during penetration. On the other hand, in the case of the TDR measurement system, only two or three wave guides are required for the instrumentation of DCP [22]. Considering the durability and simplicity of the testing equipment, the instrumentation of DCP with the wave guides is more suitable. For the evaluation of the volumetric water content of the in-situ soils with depth, Lin et al. [23] developed a TDR penetrometer by instrumenting the shaft of the penetration rod with the wave guides. In addition, for the assessment of both moisture contents and penetration resistances of in-situ soils, Vaz and Hopmans [24] developed a coiled TDR probe, and Topp et al. [25] developed a static penetration system instrumented with wave guides. However, the temperature effect on the measured relative permittivity has not been investigated, and the wave guides installed on the shaft of the penetration rod with a uniform cross-section may cause an imperfect contact with the soils if the verticality of the penetrometers is not maintained during the penetration. In this study, for effective evaluation of relative permittivity and volumetric water content in the ground, wave guides were installed on an inclined surface of the DCP apparatus to have perfect contact with the target ground. In addition, a thermo-sensor was installed near the wave guides. This is necessary because ground temperature affects ground relative permittivity. Thus, an objective evaluation of ground moisture content can be conducted by considering the temperature effect on relative permittivity.

This study develops a dynamic cone penetrometer incorporated with TDR sensors (TDCP) for evaluating the subsurface water content based on the relative permittivity compensated by the ground temperature and for characterizing strength by the penetration index. This paper documents the theoretical background of the TDR system with overall shape and function of the TDCP. In addition, this paper describes procedures and results of calibration tests for investigating the relationship between relative permittivities measured by the TDCP probe and the standard TDR probe, the temperature effects on relative permittivities, and the conversion of the penetration index evaluated by TDCP into that by standard DCP. Finally, this paper analyzes and discusses volumetric water content, ground temperature, and penetration index obtained in the field application test conducted in mountainous ground.

## 2. Time Domain Reflectometry

Time domain reflectometry (TDR) is a measurement system of electromagnetic waves propagated by a transmission line in the time domain [26]. The electromagnetic wave travels the transmission line with a velocity [27] of:

(1) $$v=\frac{c}{\sqrt{\varepsilon_{r}}}=\frac{2L}{\Delta t}$$

where v is the velocity of the electromagnetic wave travelling the transmission line, c is the velocity of the electromagnetic wave propagated in vacuum (2.998 × 10^8^ m/s), ε_r_ is the relative permittivity of the transmission line, L is the length of the transmission line, and Δt is the travel time of the electromagnetic wave travelling the transmission line. In addition, the electromagnetic wave is reflected at discontinuities of the transmission line with a reflection coefficient [28,29] as:

(2) $$R=\frac{A_{r}}{A_{i}}=\frac{Z_{2}-Z_{1}}{Z_{2}+Z_{1}}$$

where R is the reflection coefficient, A_i_ and A_r_ are amplitudes of the incident and reflected electromagnetic waves, respectively, and Z_1_ and Z_2_ are impedances of the media that make a discontinuity of the transmission line. Therefore, if relative permittivity of the transmission line is known, such as telecommunication lines, power lines, and uniform metal bars, locations of discontinuities can be accurately evaluated based on the travel time of the reflected electromagnetic waves [30,31].

**Figure 1:**
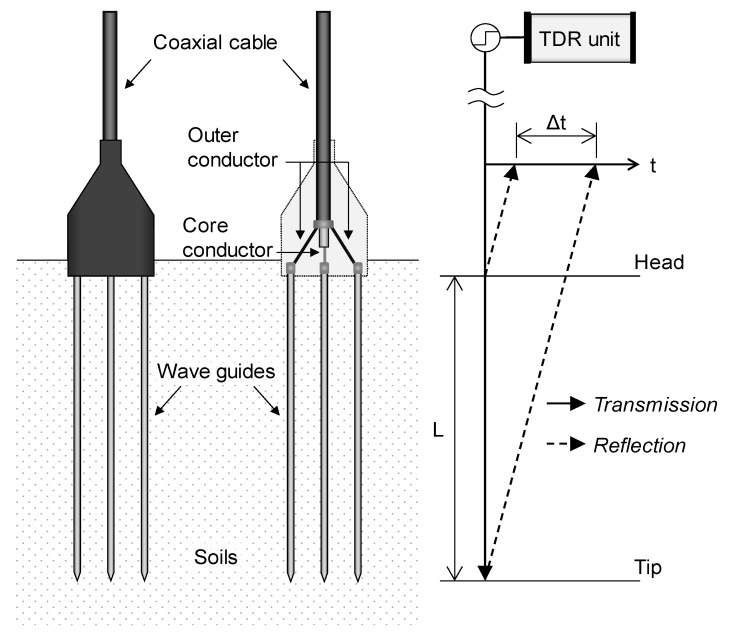
Time domain reflectometry (TDR) probe with three wave guides and propagation of the electromagnetic wave.

On the other hand, in geotechnical, geo-environmental, and agricultural practices, the relative permittivity of the transmission line is inversely calculated based on the fixed length of the transmission line and the measured travel time of the electromagnetic wave as described in Equation (1). A probe with two or three parallel wave guides is commonly adopted to evaluate the relative permittivity of the transmission line. Figure 1 shows a probe with three wave guides. The TDR unit generates an electromagnetic wave through the transmission line and gathers electromagnetic waves reflected at the discontinuities with an amplitude ratio according to the reflection coefficient R in Equation (2). Note that the head and the tip of the wave guides are discontinuities as shown in Figure 1. In the TDR probe, there are two parts of the transmission line: a coaxial cable and wave guides installed in the soil. Because the impedance and relative permittivity are uniform along the coaxial cable, the travel time of the electromagnetic wave travelling the coaxial cable is constant. At the wave guides, the electromagnetic wave propagates while generating electromagnetic field around the wave guides. Thus, the travel time of the electromagnetic wave travelling the transmission line is dependent on the relative permittivity of the soils surrounding the wave guides. In addition, based on the travel time of the electromagnetic wave (Δt) and the length of the wave guides between the head and the tip (L), the relative permittivity of the soils (ε_r_) can be calculated by using Equation (1).

**Figure 2:**
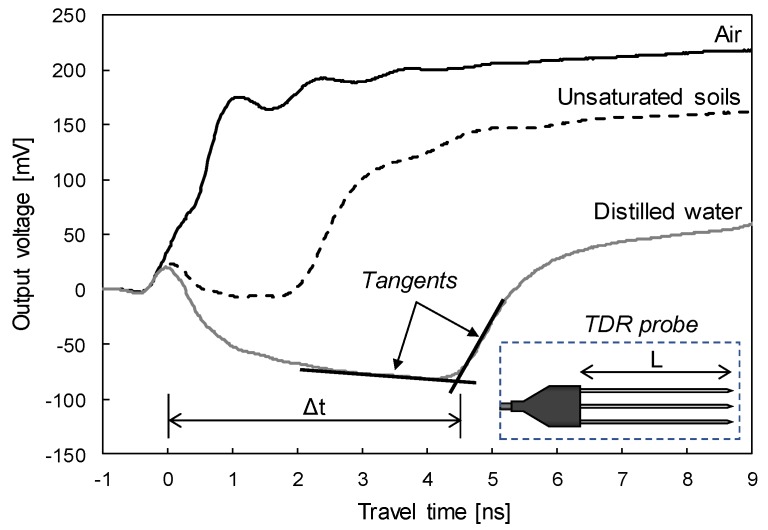
Typical TDR signals.

Figure 2 plots typical waveforms of electromagnetic waves measured in air, unsaturated soils, and distilled water. In the measured electromagnetic waves, the travel time of the electromagnetic waves can be determined by the crossing point of the tangents (see Figure 2), which is termed the tangent analysis method. Note that the tangent analysis method is known to be the most accurate method to determine the reflection point of electromagnetic waves [32,33,34].

The travel time of electromagnetic waves takes longer in the order of the media of air, unsaturated soils, and distilled water because relative permittivities of air, soil particles, and water are ≈ 1, 2~6, and 80.2 (at 20 °C) [35], respectively. As the relative permittivity of unsaturated soils is the combined relative permittivity of the three-phase soil mixture, which includes air, soil particles, and water, the travel time is located in between those of air and distilled water. In most cases, the volumetric water content of the testing soil is calculated by the quadratic or cubic equation of the relative permittivity [19] as:

(3) $$\theta_{v}=a\cdot \varepsilon_{r}{}^{3}+b\cdot \varepsilon_{r}{}^{2}+c\cdot \varepsilon_{r}+d$$

where θ_v_ and ε_r_ are the volumetric water content and relative permittivity of soils, a, b, c, and d are empirical coefficients that are dependent on the mineralogy, shape, and size of the soil particles. These coefficients are determined through a calibration process [18,24]. The quadratic or cubic equation of relative permittivity (Equation (3)) is known to have good agreement with the volumetric water contents of soils. When an error occurs between the relative permittivity and the volumetric water content estimated by Equation (3), the linear relationship between the square root of relative permittivity and the volumetric water content can be considered to reduce the error [36].

## 3. DCP Incorporated with TDR Sensors

**Figure 3:**
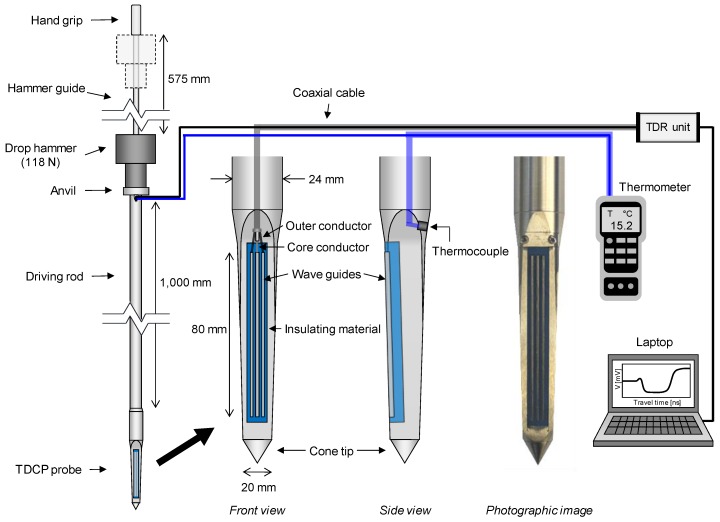
Dynamic cone penetrometer incorporated with TDR sensors (TDCP).

Although the volumetric water content of soils has good agreement with the quadratic or cubic equation of the relative permittivity measured by the TDR system, the standard TDR probe is adoptable only for laboratory tests or ground surface because the standard TDR probe is composed of two or three slender wave guides that can be easily bent or broken when penetrating the ground. Figure 3 shows a dynamic cone penetrometer incorporated with TDR sensors (TDCP) developed to evaluate the relative permittivity and volumetric water content along the depth as well as for strength characterization of the target ground.

The TDCP is composed of an anvil, extendible driving rod, and TDCP probe. The body of the TDCP probe is made of stainless steel for durability when penetrating the ground. The TDR sensor module, which is composed of insulating material (MC-Nylon) and three wave guides with a length of 80 mm, is installed on a side of the TDCP probe body as shown in Figure 3. The central wave guide is connected to the core conductor, while the other wave guides are connected to the outer conductor of a coaxial cable. On the opposite side of the wave guides, a K-type thermocouple is installed to measure the ground temperature. As the experimental procedure of the TDCP test, the TDCP is dynamically penetrated into the ground by using a hammer with a weight of 118 N at a drop height of 575 mm. During dynamic penetration of the TDCP, penetration depths and blow counts are recorded to profile the penetration index (TDCP penetration index: TPI) along the depth as:

(4) $$TPI_{}[\mathrm{mm}/\mathrm{blow}]=D_{n+1}-D_{n}$$

where D_n+1_ and D_n_ are the penetration depths at the (n + 1)th and nth blow counts, respectively. When the TDCP probe reaches the target depth for evaluating the relative permittivity and volumetric water content, a TDR unit generates the electromagnetic wave through the coaxial cable and wave guides and gathers the reflected electromagnetic wave in the time domain. The measured TDCP signals are monitored and saved with a laptop computer. In addition, ground temperatures are measured by using a K-type thermocouple and a thermometer.

## 4. Calibration Tests

### 4.1. Relative Permittivity and Volumetric Water Content by the TDCP

**Figure 4:**
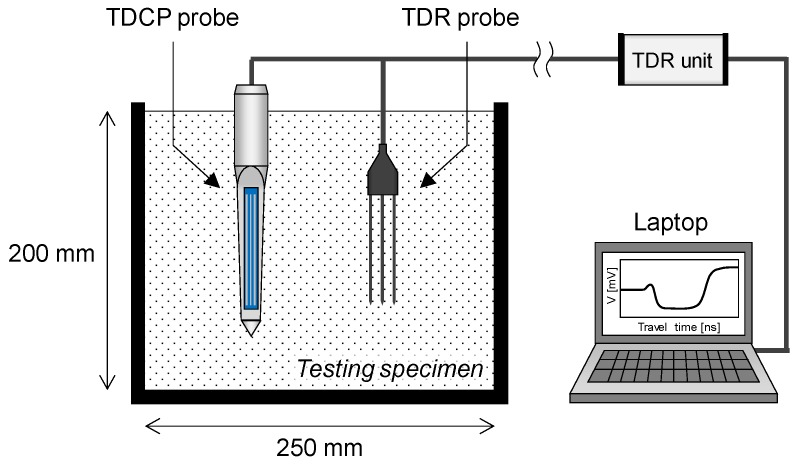
Calibrations using the TDR and TDCP probes.

**Figure 5:**
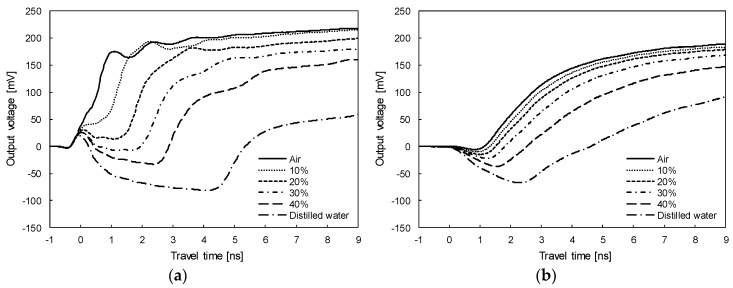
Signals at different testing samples measured using the: (**a**) TDR probe; (**b**) TDCP probe.

In the TDR measurement system, the electromagnetic wave propagates the wave guides while generating the electromagnetic field around the wave guides. Although all surfaces of the wave guides of the standard TDR probe are surrounded by soil, the wave guides of the TDCP are in partial contact with the insulating material. The insulating material includes the range of the electromagnetic field and affects the velocity of the electromagnetic wave that propagates through the wave guides. Therefore, a calibration was conducted in air, distilled water, and soil specimens with different volumetric water contents of 5%, 10%, 15%, 20%, 25%, 30%, 35%, and 40% using both TDR and TDCP probes as shown in Figure 4. In addition, the moisture contents of the testing specimens were measured after calibration to assure that the moisture contents were uniform during the calibration process. Note that temperatures of the laboratory and testing samples were kept at 20 °C, and the soils used were classified as well-graded sandy soils (SW) by the unified soil classification system (USCS) [37]. Figure 5a,b plot signals measured in air, distilled water, and soil specimens with volumetric water contents of 10%, 20%, 30%, and 40% with the two probes. The travel time is the shortest in air, increases when the volumetric water content of the soil specimen increases, and is the longest in distilled water. However, the travel times of the electromagnetic waves measured by the TDR probe and the TDCP probe differ for each testing specimen because the contacted insulating material affects the velocity of the electromagnetic wave that propagates through the TDCP wave guides.

**Figure 6:**
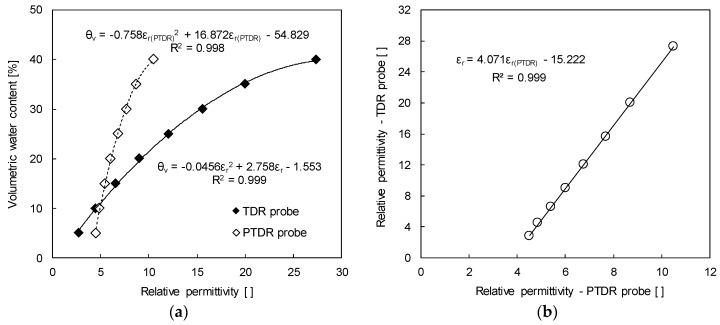
Calibration result: (**a**) Volumetric water content and the relative permittivity; (**b**) Relative permittivity measured using the TDR and TDCP probes.

The relative permittivity is calculated based on the measured travel time and is correlated with the volumetric water contents of the soil specimens as shown in Figure 6a. The volumetric water content (θ_v_) of the soil specimen is fitted with the quadratic equation of the relative permittivity evaluated by both the TDR probe (ε_r_) and the TDCP probe (ε_r(TDCP)_) as:

(5) $$\theta_{v}=-0.0456\cdot \varepsilon_{r}{}^{2}+2.758\cdot \varepsilon_{r}-1.553$$

(6) $$\theta_{v}=-0.758\cdot \varepsilon_{r(\mathrm{TDCP})}{}^{2}+16.872\cdot \varepsilon_{r(\mathrm{TDCP})}-54.829$$

In addition, the relative permittivity evaluated by the TDR probe is linearly correlated with that evaluated by the TDCP probe, with a coefficient of determination (R^2^) of 0.999 (see Figure 6b) as:

(7) $$\varepsilon_{r}=4.071\cdot \varepsilon_{r(\mathrm{TDCP})}-15.222$$

Therefore, the in-situ relative permittivity (ε_r_) and volumetric water content (θ_v_) can be reasonably evaluated by using the relative permittivity obtained from the TDCP test (ε_r(TDCP)_).

### 4.2. Temperature Effect on Relative Permittivity

In most cases, the subsurface is composed of a three-phase soil mixture such as air, soil particles, and water. Thus, the relative permittivity is greatly dependent on the volume fraction of water because the relative permittivity of the water is significantly greater than those of the other components. While relative permittivities of air and soil particles are hardly affected by temperature changes, the relative permittivity of water varies with regard to temperature change [35,38]. Accordingly, the temperature effect on the relative permittivity of the soil mixture is dependent on variations of the relative permittivity of water. Therefore, for an accurate evaluation of the volumetric water content, temperature effect on the relative permittivity of water should be investigated.

**Figure 7:**
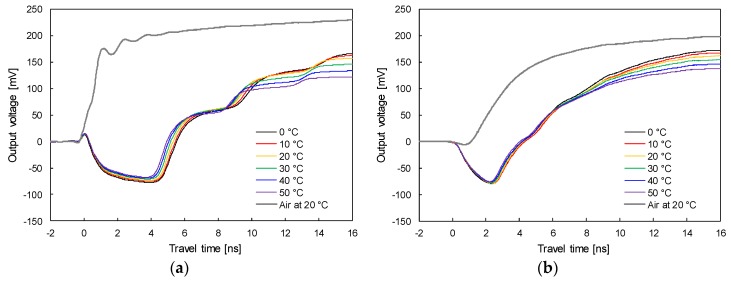
Signals in distilled water at different temperatures measured using the: (**a**) TDR probe; (**b**) TDCP probe.

To investigate temperature effects on relative permittivity, electromagnetic waves were measured using both the TDR probe and TDCP probe in distilled water at temperatures of 0 °C, 5 °C, 10 °C, 15 °C, 20 °C, 25 °C, 30 °C, 35 °C, 40 °C, 45 °C, and 50 °C. Figure 7a,b plot the signals at 0 °C, 10 °C, 20 °C, 30 °C, 40 °C, and 50 °C, measured by the TDR and TDCP probes, respectively. For both results, as the temperature of the distilled water increases, the travel time decreases.

**Figure 8:**
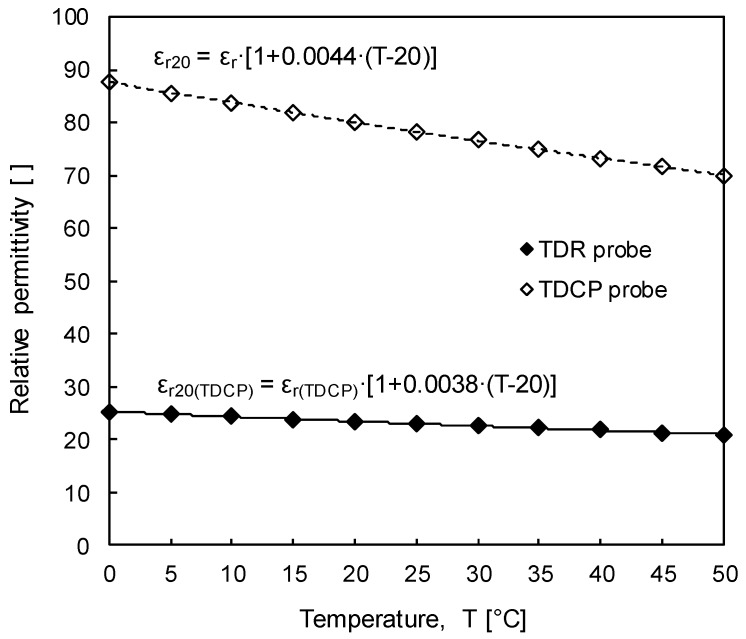
Relative permittivities of distilled water at different temperatures evaluated by using the TDR probe and TDCP probe.

In addition, with an increase in temperature, the amplitude of the reflected electromagnetic waves, which is related to the electrical conductivity of the testing specimen, decreases because an increase in temperature causes an increase in the mobility of the ions dissolved in the testing specimen. Figure 8 shows the relative permittivity according to temperature evaluated based on the travel time of the electromagnetic wave measured using both the TDR probe and TDCP probe. Relative permittivities evaluated by both TDR probe and TDCP probe at 0 °C are about 87.7 and 25.3 and gradually decrease to about 70.1 and 21.0 at 50 °C, respectively. Equations (8) and (9) describe relationships of temperature (T), relative permittivities evaluated by both standard TDR probe and TDCP probe at T °C (ε_r_ and ε_r(TDCP)_), and relative permittivities corrected at 20 °C for both standard TDR and TDCP probes (ε_r20_ and ε_r20(TDCP)_):

(8) $$\mathrm{Standard}\;\mathrm{TDR}\;\mathrm{probe}:\;\varepsilon_{20}=\varepsilon_{r}\cdot \left[1+0.0044\cdot \left(T-20\right)\right]$$

(9) $$\mathrm{TDCP}\;\mathrm{probe}:\;\varepsilon_{20(\mathrm{TDCP})}=\varepsilon_{r(\mathrm{TDCP})}\cdot \left[1+0.0038\cdot \left(T-20\right)\right]$$

Therefore, regardless of ground temperature variations, volumetric water content can be objectively evaluated by using the relative permittivity corrected at a temperature of 20°C.

### 4.3. Penetration Index Correction

The standard dynamic cone penetrometer (DCP) is composed of a driving rod with a diameter of 16 mm and a length of 1000 mm and a cone tip with an apex angle of 60° and a diameter of 20 mm. The DCP is penetrated into the ground using a hammer with a weight of 78.5 N and a drop height of 575 mm. During the dynamic penetration of DCP, penetration depths and blow counts are recorded to calculate the dynamic cone penetration index (DPI) with a unit of mm/blow for characterization of the ground strength. In order to expand the application range of the DCP, several empirical correlations between the DPI and other engineering parameters such as standard penetration test (SPT) N-value, California bearing ratio, elastic modulus, void ratio, and relative density have been suggested [12,13,14,39,40]. Although the penetration index measured by the TDCP (TDCP penetration index: TPI) can also characterize ground strength, the TPI cannot be directly applied to empirical correlations because the shape, dimensions, and driving energy of the TDCP differ from those of the standard DCP. Therefore, the correlation between TPI and DPI may be useful to estimate other engineering parameters and for strength characterization by using TPI.

**Figure 9:**
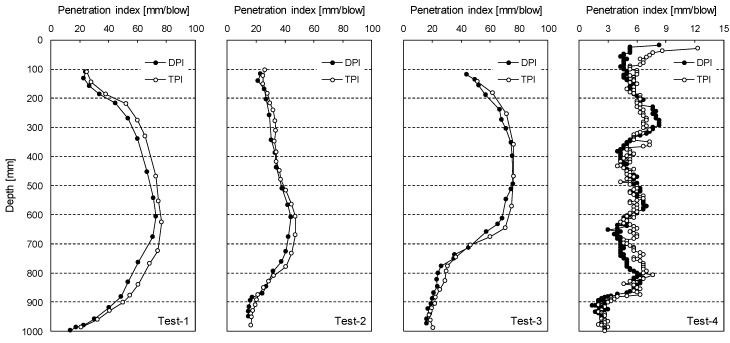
Penetration index profiles measured using the dynamic cone penetrometer (DCP) and TDCP at Test-1 to Test-4.

To investigate the relationship between TPI and DPI, penetration tests were conducted at four different grounds (Test-1 to Test-4) composed of well-graded sandy soils. Figure 9 plots the penetration indices measured at Test-1 to Test-4. TPI and DPI are marked by open circles and solid circles, respectively. For all tests, TPI is slightly greater than DPI at the same depth.

**Figure 10:**
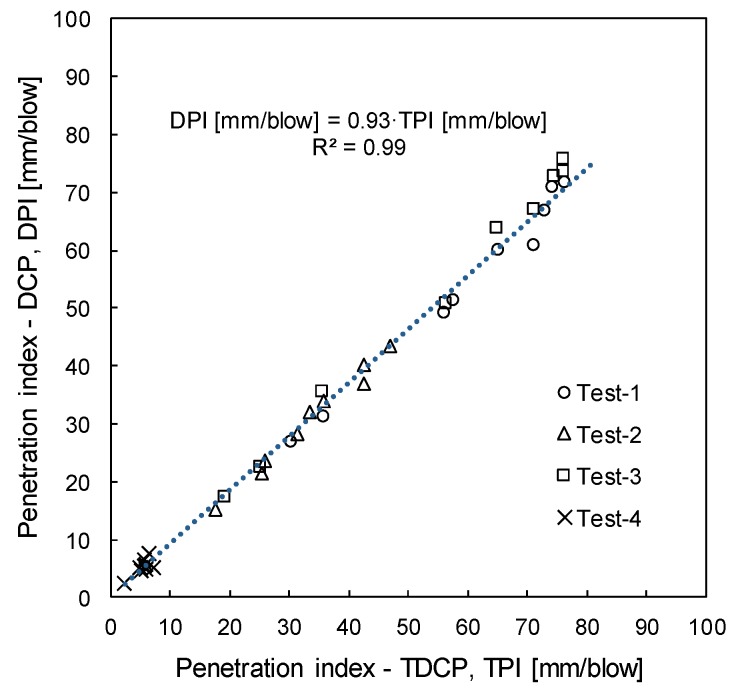
The relationship between the penetration indices measured using the DCP and TDCP.

For the comparison of measured penetration indices, TPI and DPI are averaged every 100 mm in depth. Figure 10 correlates those averaged TPIs and DPIs. The relationship between TPI and DPI is shown as:

(10) DPI[mm/blow]=0.93⋅TPI[mm/blow]

TPI is linearly correlated with DPI, with a correlation coefficient of 0.93 and a coefficient of determination (R^2^) of 0.99. Note that the correlation coefficient and the coefficient of determination were rounded to two decimal places. Therefore, the dynamic cone penetration index (DPI) by the standard DCP can be estimated by TPI. Corrected DPI may be used for estimating other engineering parameters such as the SPT N-value, California bearing ratio, elastic modulus, void ratio, and relative density.

## 5. Field Application Test

### 5.1. Experimental Setup

**Figure 11:**
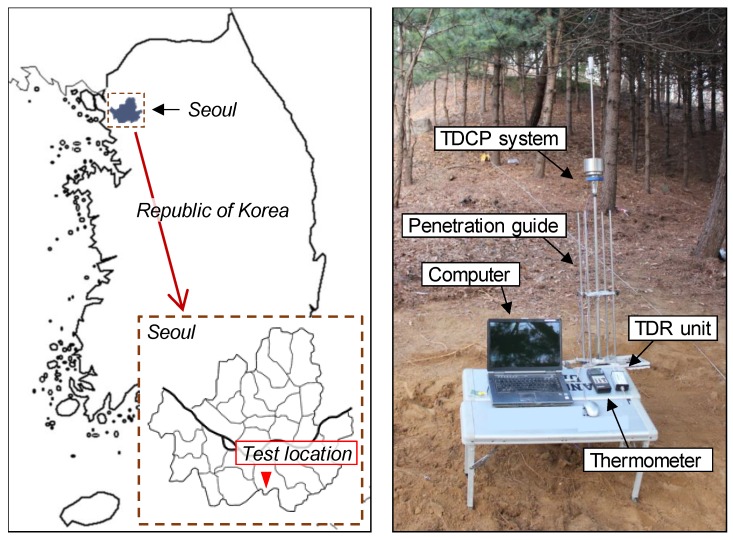
Experimental setup for the field application test.

For the application of the TDCP, a field study was conducted at a mountainous area located in Seoul, Republic of Korea, as shown in Figure 11. The target ground was located adjacent to a highly populated urban area where a landslide had occurred during the rainy season. Therefore, a portable ground investigation method needed to be applied to the target ground for evaluating the moisture content and the strength index.

At the target ground, a TDCP test was conducted for measuring the penetration index and evaluating the volumetric water content along the depth. In order to accurately maintain the TDCP vertically during dynamic penetration, a penetration guide was used as shown in Figure 11. For the calculation of penetration indices, penetration depths were recorded at each hammer blow. Electromagnetic waves and ground temperatures were measured every 100 mm in depth. A TDR unit (HL1101, Hyperlabs Inc.) was used in this study for generating the step function wave and measuring guided electromagnetic waves. These measured electromagnetic waves were monitored and saved with a laptop computer. The ground temperature was measured using a K-type thermocouple and a thermometer (4132, Control Company Inc.). In addition, soils were sampled at the testing point. Gravimetric water contents were measured at every 100 mm in depth to verify the experimental results estimated by the TDCP test.

### 5.2. Experimental Results and Analyses

**Figure 12:**
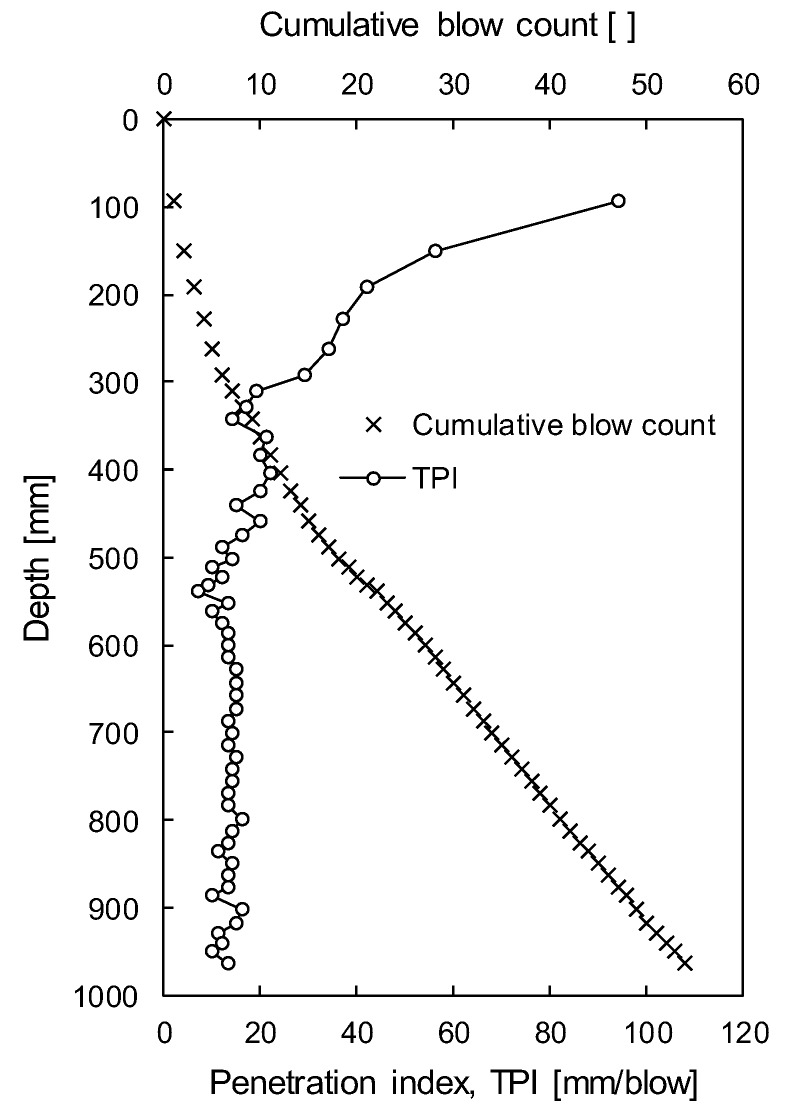
Cumulative blow count and penetration index (TPI) according to penetration depth.

During dynamic penetration of the TDCP, penetration depths were recorded at every hammer blow. Total penetration depth of the TDCP was 964 mm and total hammer blow was 54 as shown in Figure 12. In addition, TPI was calculated via Equation (4) and plotted with depth in Figure 12. The calculated TPI is 94 mm/blow near the surface and rapidly decreases to the depth of 300 mm. The TPI ranges 7~20 mm/blow at depths from 300 mm to 540 mm and converges at about 12 mm/blow below the depth of 540 mm/blow. Therefore, the target ground below the depth of 540 mm is expected to be composed of soils with relatively uniform strength.

**Figure 13:**
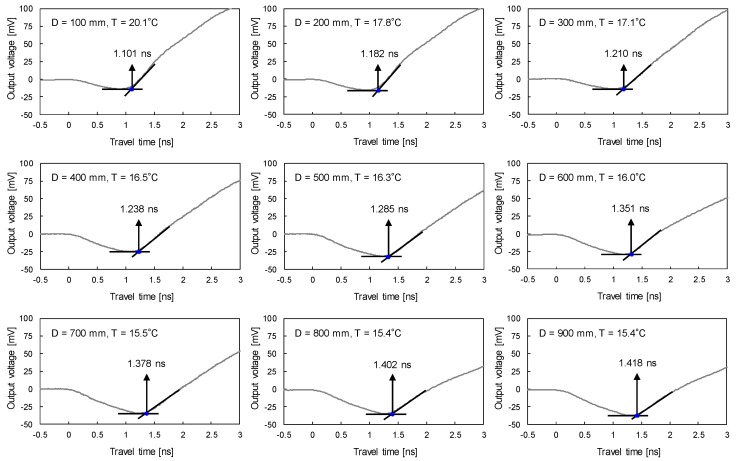
Measured TDCP signals and ground temperatures with depth.

Figure 13 shows electromagnetic waves and ground temperatures measured when the center of TDCP wave guides is located at depths from 100 mm to 900 mm with a depth interval of 100 mm. Travel times of electromagnetic waves reflected at the tip of the TDCP wave guides are determined with the tangent analysis method. For depths from 100 mm to 900 mm, the travel time of the reflected electromagnetic waves increases from 1.101 ns to 1.418 ns while the ground temperature decreases from 20.1 °C to 15.4 °C. Note that the air temperature in this study site was measured at 23.0 °C.

**Figure 14:**
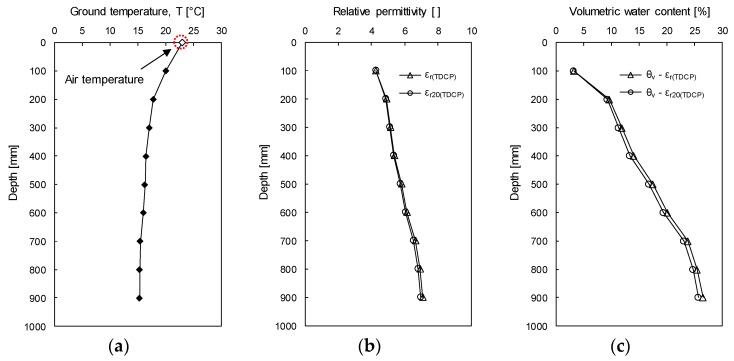
Ground temperature, relative permittivity, and volumetric water content evaluated in the field test: (**a**) Ground temperature; (**b**) Relative permittivity; (**c**) Volumetric water content.

Figure 14a profiles the measured ground temperature (T). The relative permittivity (ε_r(TDCP)_) is calculated by using the travel time of the reflected electromagnetic wave and Equation (1). In addition, the relative permittivity is corrected at a temperature of 20 °C (ε_r20(TDCP)_) based on the ground temperature profile and by Equation (9). Figure 14b plots both ε_r(TDCP)_ and ε_r20(TDCP)_ shown by open triangles and circles, respectively. At the depth of 900 mm, which shows the largest different temperature from 20 °C, ε_r(TDCP)_ and ε_r20(TDCP)_ are 7.059 and 6.936, respectively. Therefore, the error due to ground temperature is up to 1.77%. The volumetric water contents of the target ground are evaluated by using both ε_r(TDCP)_ and ε_r20(TDCP)_ via Equation (6) as shown in Figure 14c. Note that the relationship between the relative permittivity and the volumetric water content (Equation (6)) was constructed with soils sampled in the experimental study site. In the profile of volumetric water contents evaluated based on ε_r20(TDCP)_, the volumetric water content is 3.27% at the depth of 100 mm, rapidly increases along the depth, and is 25.73% at the depth of 900 mm. At the depth of 900 mm, the difference in volumetric water contents evaluated by ε_r20(TDCP)_ and ε_r(TDCP)_ is 0.77%, which is caused by the temperature effect on the relative permittivity. Although the volumetric water content was evaluated within 1 m from the ground surface, the volumetric water content significantly varies with depth. Thus, the TDCP test may be effectively used for investigating the distribution of moisture content.

### 5.3. Verification of Volumetric Water Content Estimated by the TDCP

For verification of volumetric water contents evaluated by the TDCP, soils were sampled at depths from 100 mm to 900 mm with a depth interval of 100 mm. In addition, gravimetric water contents were measured in laboratory tests. Table 1 summarizes the index properties of the field soils and gravimetric water contents measured at each depth. Although TDCP evaluated volumetric water contents, gravimetric water contents were measured from the sampled soils. Therefore, a conversion of gravimetric water content into volumetric water content is necessary for comparison with volumetric water contents evaluated by the TDCP test. Based on the three-phase system of the soil mixture, the relationship between the volumetric water content (θ_v_) and the gravimetric water content (ω) can be defined as:

(11) $$\theta_{v}=\frac{\omega \cdot G_{s}}{1+e}$$

where θ_v_ and ω are volumetric and gravimetric water contents, G_s_ is the specific gravity of soil particles, and e is the void ratio of the soil mixture. For the application of Equation (11), in-situ void ratio of field soil is required as an input property. Many researchers have suggested relationships between DPI and parameters that can be used for estimation of the void ratio as discussed in Section 4.3 Penetration Index Correction. In order to estimate the void ratio based on the DPI, relationships between the void ratio and the DPI suggested by Lee et al. [13] and Mohammadi et al. [14] were adopted. Note that soil types used by Lee et al. [13] were poorly graded sandy soil (SP), silty sand soil (SM), and SW–SM, while those used by Mohammadi et al. [14] were SW. Lee et al. [13] have suggested the relationship between void ratio, DPI, and median diameter of soil particles (D_50_) as:

(12) $$e=0.43+0.0027\cdot \frac{\mathrm{DPI}\left[\mathrm{mm}/\mathrm{blow}\right]}{D_{50}{}_{}\left[mm\right]}$$

In addition, Mohammadi et al. [14] have suggested the relationship between void ratio and DPI as:

(13) $$e=e_{\max}-1.8993\cdot \left(e_{\max}-e_{\min}\right)\cdot DPI^{-0.532}\left[\mathrm{mm}/\mathrm{blow}\right]$$

where e_max_ and e_min_ are the maximum void ratio and minimum void ratio of soils, respectively.

**Figure 15:**
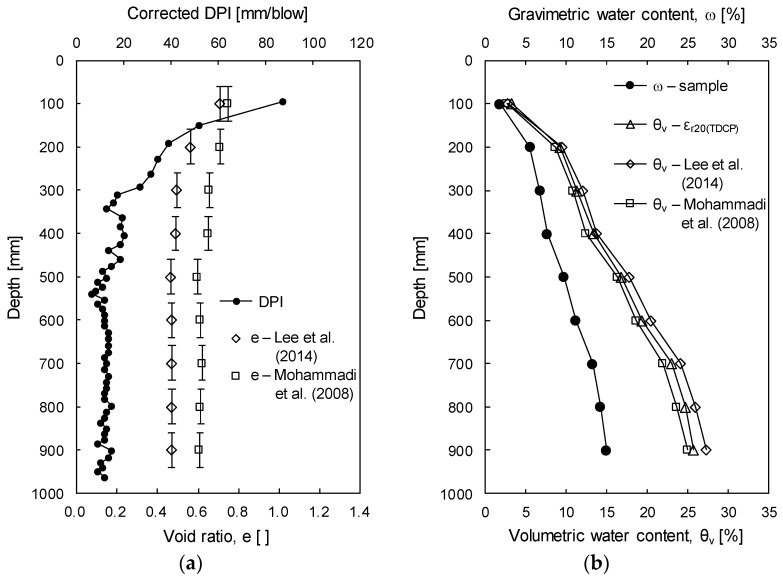
Verification of the experimental results: (**a**) Corrected dynamic cone penetration index (DPI) and void ratio; (**b**) Evaluated and estimated water contents.

To estimate the field void ratio, TPI measured in the field study was converted to DPI via Equation (10). Figure 15a plots the corrected DPI. Based on DPI, D_50_ (0.84 mm), e_max_ (0.82), and e_min_ (0.41), void ratios are calculated via Equations (12) and (13). To calculate a representative void ratio at each layer, void ratios estimated within the depths of the TDCP wave guides (80 mm at each layer) were averaged. Figure 15a plots the representative void ratios shown by open diamonds (Equation (12)) and open squares (Equation (13)). At the depth of 100 mm where DPI is greater than that at other depths, void ratios estimated by using Equations (12) and (13) are almost similar. However, the gap of the void ratios increases as the DPI decreases due to differences in experimental conditions and soils used by Lee et al. [13] and Mohammadi et al. [14]. Figure 15b plots the measured gravimetric water contents of the soil samples and volumetric water contents evaluated by the TDCP. In addition, Figure 15b plots the volumetric water contents estimated based on gravimetric water contents, void ratios, and specific gravity by using Equation (11). While volumetric water contents estimated based on gravimetric water contents and the void ratio by Lee et al. [13] are slightly greater than volumetric water contents evaluated by the TDCP, those estimated based on the volumetric water contents and the void ratio by Mohammadi et al. [14] are slightly smaller than the volumetric water contents evaluated by the TDCP. The error between the evaluated and the estimated volumetric water contents increases with depth.

**Figure 16:**
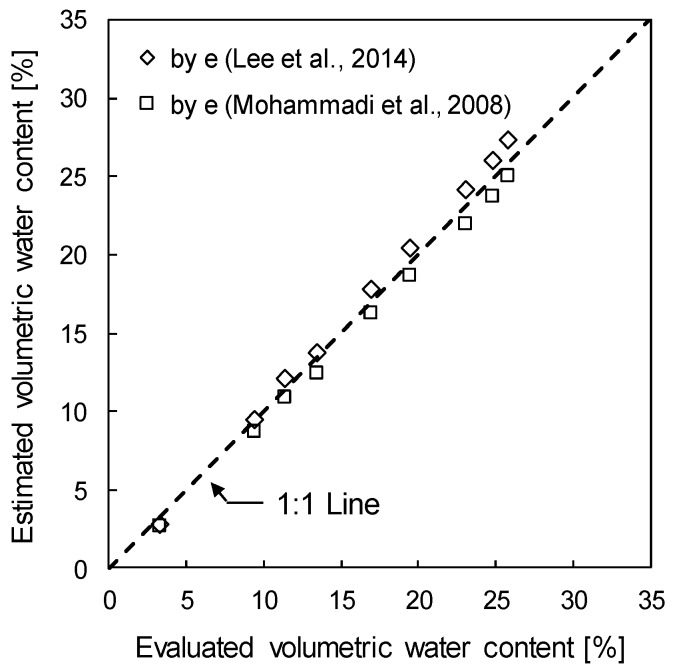
Comparison of the evaluated and estimated volumetric water contents.

Figure 16 plots the estimated volumetric water contents with volumetric water contents evaluated by the TDCP. The maximum difference in the evaluated volumetric water contents and the volumetric water content estimated by using Equations (11) and (12) is about 1.6%, while that estimated by using Equations (11) and (13) is about 0.7%. Considering the fact that void ratios are estimated without considerations of the confining stress effect on DPI or index properties of soils in the experimental study site which differ from those used by Lee et al. [13] and Mohammadi et al. [14], the differences between the evaluated and estimated gravimetric water contents are negligible. Therefore, the volumetric water content of the ground can be reasonably evaluated from the TDCP test and the penetration index measured during the TDCP test may be used for estimating engineering parameters of the target ground.

## 6. Summary and Conclusions

A dynamic cone penetrometer incorporated with TDR sensors (TDCP) was developed for the evaluation of ground volumetric water contents and for strength characterization by penetration index (TPI). The TDCP is composed of an anvil, an extendible driving rod, and a TDCP probe. The anvil, driving rod, and body of the TDCP probe are made of stainless steel for durability during the dynamic penetration into the ground. On the body of the TDCP probe, a TDR sensor module composed of insulating material and three wave guides and a K-type thermocouple are installed for evaluating the relative permittivity and ground temperature. The TDCP is dynamically penetrated into the ground using a drop hammer with a weight of 118 N at a drop height of 575 mm. During dynamic penetration, penetration depths are recorded to profile the penetration index. In addition, the relative permittivity and the ground temperature are measured at the target depth for evaluation of volumetric water contents.

For utilization of the TDCP, calibration tests were conducted. As per results of calibration tests, the volumetric water content of soils can be calculated by the quadratic equation of the relative permittivity measured by the TDCP probe. In addition, the relative permittivity measured by the TDCP probe is linearly correlated with that measured by the standard TDR probe. The temperature effect on the relative permittivity was also investigated so that an objective evaluation of the volumetric water content can be conducted. Moreover, the penetration index by the TDCP (TPI) can be converted to that by the standard DCP (DPI) using a correlation factor of 0.93 and a coefficient of determination of 0.99. Thus, the relationship between the DPI and other engineering parameters may be applicable for TPI. For the application of the TDCP, a field study was conducted with mountainous ground. As the results of the field application test of the TDCP, the TPI, ground temperature, and relative permittivity are measured and volumetric water contents compensated at a temperature of 20 °C are evaluated at depths from 100 mm to 900 mm with a depth interval of 100 mm. For the verification of the volumetric water contents evaluated by the TDCP, gravimetric water contents are converted into the volumetric water contents using the three-phase system of the soil mixture and void ratios estimated from the TPI profile. In addition, volumetric water contents evaluated by the TDCP are compared to those estimated from the soil samples. The evaluated volumetric water contents show good agreement with the estimated volumetric water contents. Thus, the volumetric water content of the subsurface can be reliably evaluated from results of the TDCP test. The TDCP developed in this study is a portable penetration testing apparatus that can characterize ground moisture content and strength with high mobility and minimal ground disturbance. Therefore, the TDCP may be effectively used for stability assessment of the ground with low accessibility, such as sloping and mountainous areas, as well as for normal subsurface.

## Figures and Tables

**Table 1:** Index properties of soils sampled in the experimental study site (Seoul, Republic of Korea).

Property	Value	
Coefficient of uniformity, C_u_ [ ]	21.14	
Coefficient of curvature, C_c_ [ ]	1.14	
Specific gravity, G_s_ [ ]	2.68	
Median diameter, D_50_ [mm]	0.84	
Soil type by USCS ^1^	SW ^2^	
Maximum void ratio, e_max_ [ ]	0.82	
Minimum void ratio, e_min_ [ ]	0.41	
Gravimetric water content, ω [%]	100 mm	1.78
	200 mm	5.52
	300 mm	6.74
	400 mm	7.63
	500 mm	9.72
	600 mm	11.22
	700 mm	13.26
	800 mm	14.26
	900 mm	15.00

^1^ USCS is the unified soil classification system. ^2^ SW is the well-graded sandy soil classified by USCS.

## References

[1] Birle E., Heyer D., Vogt N. (2008). Influence of the initial water content and dry density on the soil–water retention curve and the shrinkage behavior of a compacted clay. Acta Geotech..

[2] Wheeler S.J., Sharma R.S., Buisson M.S.R. (2003). Coupling of hydraulic hysteresis and stress–strain behaviour in unsaturated soils. Géotechnique.

[3] Chen R.H., Chen H.P., Chen K.S., Zhung H.B. (2009). Simulation of a slope failure induced by rainfall infiltration. Environ. Geol..

[4] Meyer L.D. (1981). How rain intensity affects interrill erosion. Trans. ASAE.

[5] Rahimi A., Rahardjo H., Leong E.C. (2011). Effect of antecedent rainfall patterns on rainfall-induced slope failure. J. Geotech. Geoenviron..

[6] Tu X.B., Kwong A.K.L., Dai F.C., Tham L.G., Min H. (2009). Field monitoring of rainfall infiltration in a loess slope and analysis of failure mechanism of rainfall-induced landslides. Eng. Geol..

[7] Fox G.A., Wilson G.V. (2010). The role of subsurface flow in hillslope and stream bank erosion: A review. Soil Sci. Soc. Am. J..

[8] Bengtsson T.O. (1987). The Hydrologic Effects from Intense Ground-Water Pumpagein East-Central Hillsborough County, Florida. Multidisciplinary Conference on Sinkholes and the Environmental Impacts of Karst.

[9] Hong W.T., Kang S., Lee S.J., Lee J.S. (2018). Analyses of GPR signals for characterization of ground conditions in urban areas. J. Appl. Geophys..

[10] McElvaney J., Bundadidjatnika I.R. (1991). Strength evaluation of lime-stabilised pavement foundations using the dynamic cone penetrometer. Aust. Road Res..

[11] Byun Y.H., Lee J.S. (2013). Instrumented dynamic cone penetrometer corrected with transferred energy into a cone tip: A laboratory study. Geotech. Test. J..

[12] Kim S.Y., Lee J.S. (2019). Energy correction of dynamic cone penetration index for reliable evaluation of shear strength in frozen sand-silt mixtures. Acta Geotech..

[13] Lee C., Kim K.S., Woo W., Lee W. (2014). Soil Stiffness Gauge (SSG) and Dynamic Cone Penetrometer (DCP) tests for estimating engineering properties of weathered sandy soils in Korea. Eng. Geol..

[14] Mohammadi S.D., Nikoudel M.R., Rahimi H., Khamehchiyan M. (2008). Application of the dynamic cone penetrometer (DCP) for determination of the engineering parameters of sandy soils. Eng. Geol..

[15] Fellner-Feldegg H. (1969). Measurement of dielectrics in the time domain. J. Phys. Chem..

[16] Li A.G., Yue Z.Q., Tham L.G., Lee C.F., Law K.T. (2005). Field-monitored variations of soil moisture and matric suction in a saprolite slope. Can. Geotech. J..

[17] Jones S.B., Wraith J.M., Or D. (2002). Time domain reflectometry measurement principles and applications. Hydrol. Process..

[18] Noborio K. (2001). Measurement of soil water content and electrical conductivity by time domain reflectometry: A review. Comput. Electron. Agr..

[19] Topp G.C., Davis J.L., Annan A.P. (1980). Electromagnetic determination of soil water content: Measurements in coaxial transmission lines. Water Resour. Res..

[20] Hong W.T., Jung Y.S., Kang S., Lee J.S. (2016). Estimation of soil-water characteristic curves in multiple-cycles using membrane and TDR system. Materials.

[21] Vaz C.M.P., Hopmans J.W., Macedo A., Bassoi L.H., Wildenschild D. (2002). Soil water retention measurements using a combined tensiometer-coiled time domain reflectometry probe. Soil Sci. Soc. Am. J..

[22] ASTM D6565 (2005). Standard Test Method for Determination of Water (Moisture) Content of Soil by the Time-Domain Reflectometry (TDR) Method.

[23] Lin C.P., Tang S.H., Chung C.C. (2006). Development of TDR penetrometer through theoretical and laboratory investigations: 1. Measurement of soil dielectric permittivity. Geotech. Test. J..

[24] Vaz C.M.P., Hopmans J.W. (2001). Simultaneous measurement of soil penetration resistance and water content with a combined penetrometer-TDR moisture probe. Soil Sci. Soc. Am. J..

[25] Topp G.C., Lapen D.R., Edwards M.J., Young G.D. (2003). Laboratory calibration, in-field validation and use of a soil penetrometer measuring cone resistance and water content. Vadose Zone J..

[26] Noborio K., McInnes K.J., Heilman J.L. (1996). Measurements of soil water content, heat capacity, and thermal conductivity with a single TDR probe. Soil Sci..

[27] Maxwell J.C. (1873). A Treatise on Electricity and Magnetism.

[28] Brown R.G., Sharpe R.A., Hughes W.L., Post R.E. (1973). Lines, Waves, and Antennas: The Transmission of Electric Energy.

[29] Lee J.S. Geo-Characterization using Waves—Principle to Application. Proceedings of the 19th International Conference on Soil Mechanics and Geotechnical Engineering.

[30] Lee J.S., Song J.U., Hong W.T., Yu J.D. (2018). Application of time domain reflectometer for detecting necking defects in bored piles. NDT E Int..

[31] Yu J.D., Lee J.S., Yoon H.K. (2019). Circular time-domain reflectometry system for monitoring bridge scour depth. Mar. Georesour. Geotech..

[32] Chung C.C., Lin C.P. (2009). Apparent dielectric constant and effective frequency of TDR measurements: Influencing factors and comparison. Vadose Zone J..

[33] Jiang Y.J., Tayabji S.D. (1999). Analysis of Time Domain Reflectometry Data from LTPP Seasonal Monitoring Program Test Sections.

[34] Klemunes J. (1998). Determining Soil Volumetric Moisture Content Using Time Domain Reflectometry.

[35] Haynes W.M. (2014). CRC Handbook of Chemistry and Physics.

[36] Gnatowski T., Szatyłowicz J., Pawluśkiewicz B., Oleszczuk R., Janicka M., Papierowska E., Szejba D. (2018). Field Calibration of TDR to Assess the Soil Moisture of Drained Peatland Surface Layers. Water.

[37] ASTM D2487 (2017). Standard Practice for Classification of Soils for Engineering Purposes (Unified Soil Classification System).

[38] Roth K., Schulin R., Flühler H., Attinger W. (1990). Calibration of time domain reflectometry for water content measurement using a composite dielectric approach. Water Resour. Res..

[39] Livneh M., Ishai I. The Relationship Between in Situ CBR Test and the Various Penetration Tests. Proceedings of the First International Conference on Penetration Testing.

[40] Webster S.L., Grau R.H., Williams R.P. (1992). Description and Application of Dual Mass Dynamic Cone Penetrometer.

